# The impact of state and trait general and social anxiety on theory of mind

**DOI:** 10.1038/s41598-026-36718-5

**Published:** 2026-03-05

**Authors:** C. Foulds, V. Khudiakova, A. D. R. Surtees

**Affiliations:** University of Birmingham, Birmingham, UK

**Keywords:** Theory of mind, Anxiety, General anxiety, Social anxiety, State anxiety, Trait anxiety, Egocentrism, Psychology, Human behaviour

## Abstract

Theory of mind is an important skill to function in society. There is evidence that anxiety can impair the ability to use this skill effectively; however, current research is inconclusive, uses heterogeneous measures of theory of mind, and lacks distinction between anxiety types, despite these being known to present differently in clinical populations. False belief tasks are standard practice for assessing theory of mind in children and autistic participants and have also been found to adequately detect theory of mind differences in neurotypical adults yet are not systematically used throughout the current literature. Given that egocentrism reflects difficulty inhibiting one’s own perspective, a core component of theory of mind, the present study examined egocentric bias using a false belief task. One hundred and sixty-eight participants completed the Generalised Anxiety Disorder-7 (GAD-7) and the Social Phobia Inventory (SPIN) prior to being randomly allocated to a general anxiety, social anxiety, or neutral mood condition. Following mood manipulation, participants completed a false belief task with either privileged knowledge or no knowledge. A 3 × 2 ANOVA found no significant main or interaction effects of general or social anxiety and false belief knowledge on performance, despite a reported increase of anxiety in the anxiety groups. There were also no significant correlations between trait general or social anxiety on theory of mind. The findings from this study do not support the previous evidence that anxiety increases egocentrism. Future studies should continue to distinguish systematically between state, trait, general, and social anxiety, and use reliable theory of mind measures.

**Supplementary Information:**

The online version contains supplementary material available at 10.1038/s41598-026-36718-5.

## Introduction

In a fundamentally social world, developing social relationships and understanding others is an important skill. One key component of this is theory of mind, which is the ability to attribute mental states, such as beliefs, desires, and intentions to others^1^. Many factors can affect this ability, including emotions such as anxiety, which exists in a variety of categories (general, social), and can be experienced intermittently (state) and more persistently (trait). Some emotions may improve our theory of mind ability and others may reduce it. It is currently not clear whether state, trait, general, or social anxiety differ in their impact on theory of mind. Given the prevalence of anxiety disorders^2^, and the need for an improved understanding and treatment of conditions that negatively impact social interactions, it is important to understand how theory of mind processes, such as belief reasoning, vary in relation to different sorts of experiences of anxiety.

### Theory of mind

Social interactions are a ubiquitous aspect of human life^3–5^. Our world is inherently social; to survive and thrive as individuals, as well as increase social capital, effectiveness at socialising with others is crucial^6^. Understanding our own behaviours, intentions, and mental states as well as those of others is necessary for the effective management of social encounters. Various definitions have been used for these processes, most often “mentalizing” and “theory of mind”^1^. Quesque et al. (2024)^7^ distinguish mentalizing as the cognitive process of attributing mental states to others, whereas theory of mind refers to the broader theoretical construct that encompasses this process and its role in explaining and predicting others’ behaviour.

Given the breadth of purposes theory of mind serves, it is not a unitary ability^8^. It has therefore been tested via a range of different measures^9^. These can broadly be considered under subcategories of tasks that require participants to infer an actor’s beliefs, perspectives, desires, or intentions, and tasks that require participants to infer emotions^10^. All theory of mind tasks involve cognitive processing, so this distinction reflects the type of mental state assessed rather than separate abilities. Amongst the most popular cognitive theory of mind measures of false belief reasoning, which require participants to reason that a participant will (wrongly) act on their beliefs to make decisions when they are ill-informed^11^. In the current study, we use the *Vicki’s violin task*^12^, which adapts a classical false belief task to make it effective for healthy adults, who are expected to perform at ceiling on standard tasks. In this task, participants learn about a character whose violin is moved when they are out of the room and have to judge how likely it will be that Vicki will search for it where she left it, or in its new, true location. Birch and Bloom (2007)^12^ found that adults who knew about the true location of the violin over-estimated the likelihood that Vicki would correctly search in its true location. They argued that this reflected a “curse of knowledge,” such that participants’ own knowledge wrongly impacted their judgement of Vicki’s likely behaviour. Alternative cognitive measures of theory of mind require reasoning about faux pas^13^ or understanding why a person has acted in an unexpected way^14^. Common affective theory of mind tasks often require participants to judge complex emotions of another person, such as in the Reading the Mind in the Eyes task^15^.

Traditional ways of thinking about theory of mind emerged from literature in children and non-human animals^16^ and have tended to focus on broad group differences, neglecting that while different people vary in theory of mind ability, this can also vary within an individual from moment to moment. Individual differences in theory of mind ability have been well researched in terms of age^17^, culture^18^, language ability^19^, autism, visual impairments, and auditory impairments^20^. Todd and Tamir (2024)^21^ include some of the more transitory factors in their review of factors that that can lead to an over-reliance on self-information when making inferences about another’s mental state, a process often referred to as egocentrism, including incidental emotions (emotions brought about by something unrelated to the main task). Importantly, while egocentrism can impair theory of mind performance, it is not synonymous with theory of mind performance. Converse et al. (2008)^22^ found that incidental emotions that are positive in valence increase egocentrism (an over-reliance on self-information when making inferences about another’s mental state), yet Yip and Schweitzer (2019)^23^ found a negatively valanced emotion, anger, to also reduce theory of mind accuracy. Other studies have found that emotions high in uncertainty, such as anxiety, are also more likely to increase egocentrism^6,24,25^, which may in turn impair theory of mind performance under certain conditions.

### Anxiety and theory of mind

Anxiety is a future-oriented^26^, pervasive and valuable emotion that indicates potential threat and is designed to promote action to reduce susceptibility^27^, such as seeking and using advice^28^. It has been described as negative in valence and high in physiological arousal^29^. State and trait anxiety differ; the former is a common and momentary emotion, and the latter reflects a chronic predisposition. Similarly, social and general anxiety differ; Khdour et al., (2016)^30^ note the differences between social anxiety disorder (SAD) and generalised anxiety disorder (GAD) symptomology, including in cognitive domains. While SAD has been found to produce impairments in attentive, executive, and visuo-spatial functions^31^, no correlation between cognitive deficits and GAD have been reported, although there has been limited investigation into cognitive function in GAD in particular^31,32^. Outside of cognitive domains, SAD and GAD are also distinct in physiological arousal, emotional reactivity, and interpersonal patterns^33,34^.

Surtees et al., (2024)^32^ propose that anxiety increases egocentrism, as it creates a motivation to reduce uncertainty, which results in an over-reliance on our own thoughts and beliefs to predict those of others. This is in line with the broader theory that specific emotions impact specific decision-making tendencies^24^. However, there is limited and inconsistent literature regarding the impact of state anxiety, both general and social, on theory of mind. Some of these inconsistencies may have arisen from varied methodological approaches to measuring theory of mind, either through measuring different aspects of theory of mind (e.g., cognitive vs. affective) or through the level of control provided to compare social and non-social processes. Further, some differences may be a result of researchers not distinguishing between general and social anxiety.

Using spatial and conceptual perspective-taking tasks to measure theory of mind, along with an autobiographical writing task to induce state anxiety that did not distinguish between general and social anxiety, Todd et al. (2015)^6^ found increased egocentrism in anxious participants compared to anger, disgust, and neutral mood conditions. Similarly, Todd and Simpson (2016)^35^ used a visual perspective-taking task and an autobiographical writing task and again did not distinguish between general and social anxiety. They found that anxiety, relative to anger and neutral feelings, impaired ability to use theory of mind with other people but notably not non-human subjects, highlighting a link between social aspects of cognition and a reduced theory of mind ability. Dyer et al. (2021)^36^, on the other hand, distinguish between both state and trait, and general and social anxiety. In their study, state anxiety was induced through inhalations of 7.5% carbon dioxide enriched air, which they compare to the physiological and physical symptoms experienced in GAD and note that anxiety induction methods that incorporate a social element may produce different outcomes. The effects of this on emotion recognition task performance (emotion recognition is often closely related to theory of mind^37^ found high state anxiety to reduce emotion recognition accuracy. They also found that trait anxiety did not have an impact or moderate the impact of state anxiety on emotion recognition despite previous findings.

The findings from Dyer et al. (2021)^36^ highlight that there is also a lack of clarity at the trait level. A meta-analysis by Baez et al. (2023)^38^ found that individuals with SAD performed worse than healthy controls on theory of mind tests but were unable to draw any conclusions on GAD as only two studies met inclusion criteria. The outcomes of the included SAD studies varied in their findings. Some studies reported that social anxiety disorder can lead to excessive theory of mind use^39,40^. Although this may be context dependent^41^, other studies have suggested that SAD is negatively associated with theory of mind ability^42^. Furthermore, Lenton-Brym et al. (2018)^43^ found no significant differences on a social cognition task when they grouped participants based on their scores on the Social Phobia Inventory (SPIN) into sub-clinical high socially anxious and low socially anxious. For GAD, Zainal and Newman (2018)^44^ found that individuals with GAD performed better than controls at cognitive reasoning theory of mind tasks when induced with worry compared to when given a relaxation exercise, but also performed better when worried and presented with negative social stimuli compared to controls.

Baez et al. (2023)^38^ note that the substantial heterogeneity in outcomes across the included SAD studies may be due to the diversity of tests used to assess theory of mind; of the 18 SAD studies included in this meta-analysis, 13 different theory of mind tasks were used. These tasks vary not only in format but also in the specific components of theory of mind they target. Broadly, these tasks can be divided into those assessing cognitive theory of mind (e.g., false belief reasoning) and those assessing affective theory of mind (e.g., emotion recognition tasks such as the Reading the Mind in the Eyes Test). This distinction is relevant in the context of anxiety, as different components of theory of mind may be differentially affected. They conclude that further studies with large homogeneous samples were needed to better understand the factors that influence social cognition outcomes in both SAD and GAD. Quesque and Rossetti (2020)^45^ identify two criteria that a task must meet to measure theory of mind: (1) the task must involve attributing mental states to others (mentalizing), and (2) participants must maintain a distinction between their own and others’ mental states, which they label the “non-merging” criterion. While they note that many tasks do not meet these criteria, they determine that false belief tasks are an adequate method of measuring theory of mind. False belief tasks require participants to infer another person’s false belief about a particular scenario where they may or may not have privileged information about the scenario. Birch and Bloom (2007)^12^ suggest that a curse of knowledge bias in false belief reasoning can detect deficits in adult participants, i.e., if specific knowledge about an outcome increases egocentrism, and the study by Converse et al. (2008)^22^ has shown that mood manipulation can affect participants’ responses on this task.

## Current study

The current study attempted to address the lack of clarity on distinguishing how state and trait, and general and social anxiety impact theory of mind. General and social state anxiety were manipulated, and general and social trait anxiety were measured. It also attempted to address the inconsistencies and oversights in measuring theory of mind by using a typical measure of theory of mind that has been well established in the literature. A widely used false belief task^1^ measured theory of mind in participants that were randomised to either a general anxiety, social anxiety, or neutral condition. In this task, participants have to predict the behaviour of a character based on their outdated false belief. The key condition requires them to ignore their own privileged knowledge, thus providing a direct measure of egocentrism. It was predicted that the anxiety conditions would demonstrate more egocentrism in their theory of mind judgements when compared to the neutral condition, and that the social anxiety condition would perform worse than the general anxiety condition.

## Method

Methods were pre-registered on the Open Science Framework (https://osf.io/zc3pf/). Ethical approval for the study was obtained from the University of Birmingham Science Technology Engineering and Mathematics Ethics Committee. All methods were performed in accordance with the relevant guidelines and regulations.

The study followed a 3 × 2 between-subjects experimental design. Participants were pseudo-randomly assigned to one of three mood conditions (general anxiety, social anxiety, neutral) and one of two knowledge conditions (no knowledge, privileged knowledge).

### Participants

A G*power analysis based on Converse et al., (2008)^22^, to give 80% power to detect a medium effect size (ηp^2^ = 0.06) at α < 0.05, returned a suggested sample size of 155. One hundred and sixty-eight participants were recruited, exceeding the minimum sample size required to detect a medium effect size. Participants were split evenly (56 in each group) across the general anxiety, social anxiety, and neutral mood conditions. Half of each group received privileged knowledge and half received no knowledge for Vicki’s violin task (see below). Most of the participants were female and white. Mean age of participants was 19.06 (SD 1.38), with a range of 18–32. Demographic information across conditions can be found in Table 1. Participants received research credits for taking part. Participants were ineligible to take part if they were under 18, had a diagnosed psychiatric condition and/or neurodevelopmental disorder, their English language proficiency was below that required to perform the experiment, or if they were already participating in a trial using the same or similar protocol.

**Table 1:** Demographic information by Condition.

	Condition												Total	
	General anxiety				Social anxiety				Neutral					
	No knowledge		Privileged knowledge		No knowledge		Privileged knowledge		No knowledge		Privileged knowledge			
	*M*	*SD*	*M*	*SD*	*M*	*SD*	*M*	*SD*	*M*	*SD*	*M*	*SD*	*M*	*SD*
Age	19	0.90	19	0.86	18.96	0.84	18.89	0.99	18.96	0.69	19.54	2.80	19.06	1.38
	*N*	*%*	*N*	*%*	*N*	*%*	*N*	*%*	*N*	*%*	*N*	*%*	*N*	*%*
Gender														
Female	24	85.7	24	85.7	24	85.7	24	85.7	23	82.1	25	89.3	144	85.71
Male	2	7.1	4	14.3	4	14.3	4	14.3	5	17.9	3	10.7	22	13.10
Non-binary	2	7.1	-	-	-	-	-	-	-	-	-	-	2	1.19
Ethnicity														
White	14	50	14	50	13	46.4	15	53.6	19	67.9	12	42.9	87	51.79
Black	6	21.4	1	3.6	3	10.7	2	7.1	4	14.3	3	10.7	19	11.31
Asian	6	21.4	11	39.3	10	35.7	8	28.6	2	7.1	9	32.1	46	27.38
Mixed	1	3.6	2	7.1	-	-	2	7.1	3	10.7	4	14.3	12	7.14
Other	1	3.6	-	-	2	7.14	1	3.6	-	-	-	-	4	2.38
*N* total	28		28		28		28		28		28		168	

Note. Aggregated scores were used for ethnicity.

### Procedure

On arrival at the lab, a QR code was scanned by participants to access and complete the information sheet and consent form via Qualtrics, along with the GAD-7 and the SPIN to measure trait general and social anxiety. Participants were then pseudo-randomly allocated to one of the general anxiety, social anxiety, and neutral conditions, and given the relevant written instructions (detailed below) for the respective mood manipulation task. This was followed by a mood manipulation check, Vicki’s violin task (described below), and the debrief form.

### Trait anxiety measures

The GAD-7 is a brief, validated tool for measuring generalised anxiety and its severity, and has shown excellent internal consistency (α = 0.92), good test-retest reliability (intraclass correlation = 0.83) and good criterion, construct, factorial, and procedural validity^46^. Participants are asked to consider the extent to which they have experienced seven items over the past 2 weeks. Responses range from ‘Not at all’ to ‘Nearly every day’. A score between 5 and 9 indicates mild general anxiety, 10–14 moderate general anxiety, and 15–21 severe general anxiety. In the current sample, the GAD-7 demonstrated good internal consistency (α = 0.82).

The SPIN is a 17-item, validated self-rating scale assessing fear, avoidance, and physiological symptoms of social anxiety. It has shown good test-retest reliability (*r* =.78-0.89.89.89), excellent internal consistency for the full scale (α = 0.94), and good convergent validity^47^. Participants are asked to rate the extent to which each statement applied to them over the past week. Responses range from ‘Not at all’ to ‘Extremely’. A score of 21–30 indicates mild social anxiety, 31–40 moderate, 41–50 severe, and above 50 very severe social anxiety. In the present study, the SPIN showed excellent reliability (α = 0.91).

### Mood manipulation

Each of the mood manipulation tasks included a relevant writing activity as well the prospect of a future follow-up activity, reflecting the future-oriented nature of anxiety^26,48–51^. This procedure has been shown to induce anxiety in healthy participants^52^. For consistency, the neutral condition also received a prospective future follow-up task.

Participants in the social anxiety condition were asked to complete an autobiographical writing task of a time at which they were worried before or during a social event: “Think about a time you felt anxious about having to speak in public. This might be a presentation or speech. When you have decided on a memory, write about it for 5 minutes. You are encouraged to think about thoughts that crossed your mind, how other people looking at you made you feel, and any physical sensations you experienced.” Participants were also told that they will have to present what they have written: “At the end of these tasks, you will be asked to present on what you wrote for a further 5 minutes.”

Participants in the general anxiety condition were asked to complete a writing task, in which they describe a time when they felt worried about an exam: “Think about a time you had to take an important or difficult test/exam. The memory you think of should be of a time you were feeling anxious about taking the test/exam. When you have thought of the memory, spend 5 minutes recounting this experience. You are encouraged to think about how you felt, worries you had, and any physical sensations you experienced.” They were also informed that following the writing task, they will be given a test that will be marked: “At some point throughout these tasks, you will be given a test that will be marked and your result fed back to you.”

Participants in the neutral condition were asked to write about the last items they purchased from the grocery store, as used by Todd et al., 2015^6^: “Think about what you bought the last time you went shopping. You will have 5 minutes to write about the items you bought. You are encouraged to think about the items in detail; how much they cost, if you had bought them before, etc.” Participants were informed that after completing the writing task, they will be asked to engage in a prospective neutral task: “Following this you will be asked to look at pictures of items from a shopping list.”

### Manipulation check

Following the writing tasks, all participants were asked to complete a self-report questionnaire on their mood. This consisted of a 7-item Likert scale ranging from “Not at all” to “Very much so” on how strongly they felt anxious, nervous, tense, calm, indifferent, neutral, unemotional, alert, aroused, energetic, and excited (adapted from Todd et al., 2015^6^.

### False belief task

**Fig. 1:**
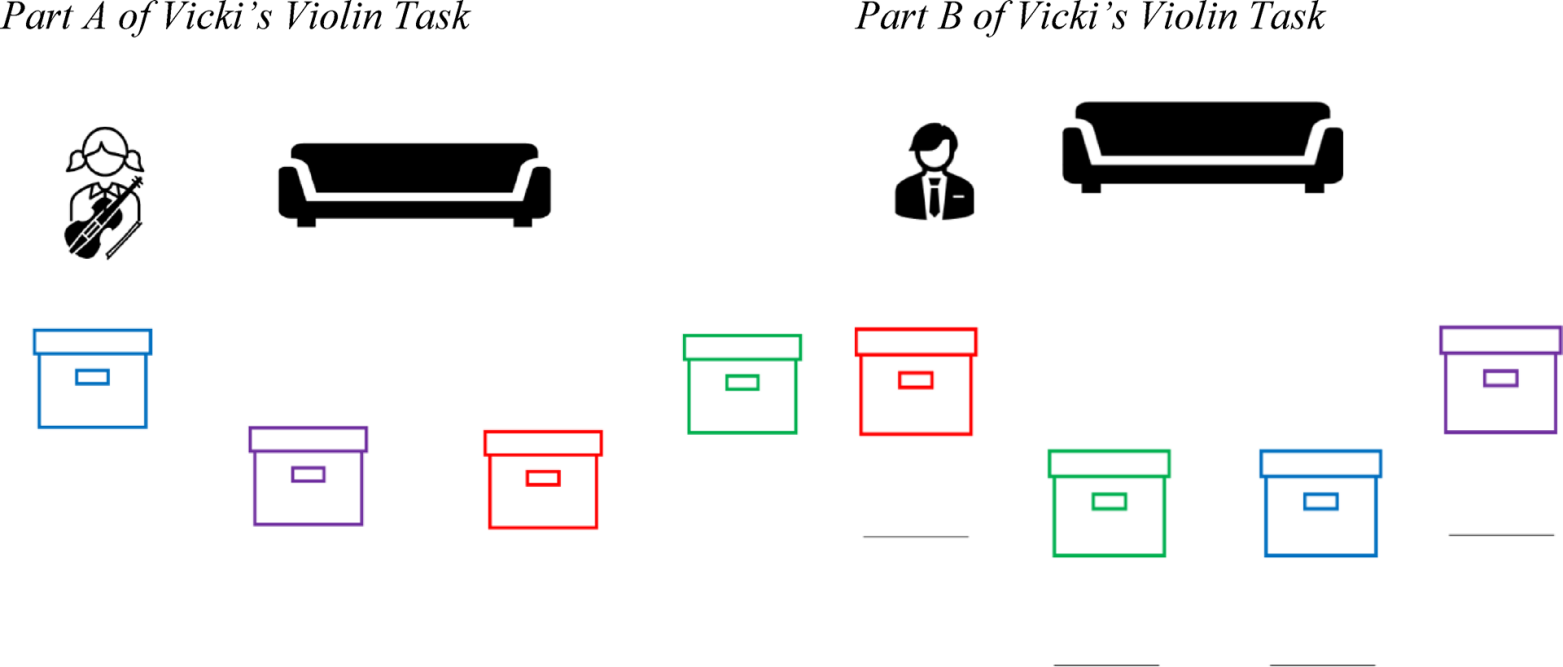
Vicki’s violin task.

Following the writing task and manipulation check, participants from each condition then completed ‘Vicki’s violin’ task as shown in Fig. 1 (adapted from^22^. In the violin task, participants are shown scenarios in which an agent places a violin in one location and leaves the scene. While the agent is away, the violin is moved to a new location. Participants are then asked to predict where the agent will search for the violin upon returning. Participants must engage in theory of mind to incorporate their knowledge of the agent’s outdated (and false) belief about the violin’s location into their predictions. Participants were randomly allocated either to a privileged knowledge condition or to receive the same knowledge that Vicki had. Both conditions receive the following text and visual: “This is Vicki. She finishes playing her violin and puts it in the blue container. Then she goes outside to play.”

The privileged knowledge group read: “While Vicki is outside playing, her sister, Denise, moves the violin to the red container.” The no knowledge condition received the same knowledge as Vicki, which read: “While Vicki is outside playing, her sister, Denise, comes into the room.” Both conditions receive the following text and image: “Then, Denise rearranges the containers in the room until the room looks like the picture below. When Vicki returns, she wants to play her violin. What are the chances Vicki will first look for her violin in each of the below containers? Write your answers in percentages in the space below each box.” The key dependent variable is the estimated likelihood, as a percentage, that the participant attributes to the red box. In the privileged knowledge condition, if participants estimate a higher likelihood of Vicki looking in the red box compared to the no knowledge condition, this demonstrates egocentrism. The no knowledge condition acts as a control, in that the percentage estimates reflect how often participants identify the red box on the basis of it being moved alone. Responses on the false belief task that were more than 3 SDs from the overall mean were removed from the analysis, as described in Converse et al. (2008)^22^. Though the task has no normatively correct response, the task acts as a measure of egocentrism on a group-level. Evidence of higher percentage estimates in the privileged knowledge condition, show the incorrect application of self-knowledge in appraising another’s. This measure can also be considered to be analogous to a measure of theory of mind performance, in the sense that applying irrelevant self-knowledge deters from logical reasoning.

Once participants had completed the mood manipulation and false-belief tasks, they were presented with a debrief form that requested they do not share any details with their peers, as well as information regarding services that can be accessed for support with any prolonged anxiety that might be experienced.

### Statistical analyses

SPSS 29.0 was used to conduct statistical analyses. The dependent variable was recorded as a scaled percentage of what participants recorded as the likelihood of Vicki looking in the red box. For most participants, this reflected the percentage score they reported. Due to some participants providing a total percentage (for all boxes) that did not equal 100%, a scaled percentage value for the dependent variable was calculated by dividing 100 by the total percentage participants gave (for all boxes) and then multiplying this by the original red percentage value. Analyses of variance (ANOVA) and chi-square tests were carried out to assess for any differences between mood conditions and knowledge conditions in terms of age, gender, and ethnicity. ANOVA were also carried out to assess for any differences between group scores on the GAD-7 and SPIN, as well as on the manipulation check variables, with Scheffe post hoc tests. A 3 × 2 ANOVA was conducted to find any main or interaction effects between the two independent variables of mood condition (general anxiety, social anxiety, and neutral) and knowledge condition (no knowledge and privileged knowledge) and their impact on the dependent variable. Where relevant, effect sizes are reported as partial eta squared (η²). Although data were not normally distributed, an ANOVA was still conducted as these are typically considered robust to variations from non-normal distributions, particularly when group sizes are balanced^53^ and with sample sizes exceeding 30^54^. The current data met both criteria. However, for completeness, the core analysis looking at the impact of were repeated with bootstrapping, with no impact on the findings (see supplementary materials). Spearman’s r correlations were performed for exploring the effects of trait anxiety on false belief task response.

## Results

### Descriptive statistics

There was no significant main effect of mood condition, F(2, 166) = 0.83, *p* =.54, or knowledge condition, F(1, 166) = 0.59, *p* =.44, and no significant interaction between the two, F(2, 166) = 0.90, *p* =.41, indicating that there were no age differences between conditions, either in terms of mood or knowledge. There was no significant difference between the gender of participants across the three mood conditions (χ2(4) = 4.26, *p* =.37). Within each of the mood conditions, there was also no difference between the gender of participants across knowledge conditions (General, χ2(2) = 2.67, *p* =.26; Social, χ2(2) = 0, *p* = 1; Neutral, χ2(2) = 0.58, *p* =.45). There was no significant difference between the ethnicity of participants across the three mood conditions (χ2(34) = 29.93, *p* =.67). Within each of the mood conditions, there was also no difference between the gender of participants across knowledge conditions (General, χ2(12) = 12.15, *p* =.43; Social, χ2(13) = 11.49, *p* =.57; Neutral, χ2(12) = 14.38, *p* =.28). There were also no significant differences between the combined conditions and scores on the GAD-7 or SPIN, *F*(5, 166) *=* 1.53, *p* =.22. Means and SDs for each group are shown in Table 2, demonstrating that the groups were equally matched for trait anxiety.

**Table 2:** Means and standard deviations of scores on the GAD-7 and SPIN by study Condition.

	Condition													
	General anxiety				Social anxiety				Neutral					
	No knowledge		Privileged knowledge		No knowledge		Privileged knowledge		No knowledge		Privileged knowledge		Total	
	*M*	*SD*	*M*	*SD*	*M*	*SD*	*M*	*SD*	*M*	*SD*	*M*	*SD*	*M*	*SD*
GAD	5.29	3.57	6.00	3.80	5.11	2.97	6.86	4.21	6.50	4.26	5.86	4.32	5.93	3.87
SPIN	19.04	10.59	24.54	12.46	21.14	9.93	24.07	12.63	19.86	11.79	19.32	10.96	21.33	11.48

On the GAD-7, 66 participants scores fell within the minimal range, 75 within the mild range, 21 within the moderate range, and 6 within the severe range. On the SPIN, 86 participants scored within the minimal range, 42 within the mild rage, 32 within the moderate range, 6 within the severe range, and 2 within the very severe range.

### Manipulation check

An ANOVA looking at differences between each of the manipulation check items and the three mood conditions found there were significant differences between condition (general anxiety, social anxiety, neutral) and the extent to which participants reported feeling anxious *F*(2, 165) *=* 15.41, *p* <.001, η² = 0.16, General = Social > Neutral, nervous *F*(2, 165) *=* 9.77, *p* <.001, η² = 0.11, General = Social > Neutral, tense *F*(2, 165) *=* 13.35 *p* <.001, η² = 0.14, General = Social > Neutral, calm *F*(2, 165) *=* 6.71, *p* =.08, η² = 0.06, General = Social < Neutral, and neutral *F*(2, 165) *=* 7.27, *p* <.001, η² = 0.08, General = Social < Neutral. This suggests that the manipulation induction was successful, in that the anxious groups reported increased anxiety with similar intensity for both social and non-social situations. No other mood manipulation items were significantly different between mood conditions. Results for each mood manipulation check item can be found in Table 3.

**Table 3:** Means, standard Deviations, and analysis of variance (ANOVA) results of manipulation check Items.

	Condition						ANOVA		Scheffe post-hoc		
	General anxiety		Social anxiety		Neutral				General v social	General v neutral	Social v neutral
	*M*	*SD*	*M*	*SD*	*M*	*SD*	*F*	*p*	*p*	*p*	*p*
Anxious	2.96	1.35	2.95	1.21	1.82	1.18	15.41	< 0.001	1	< 0.001	< 0.001
Nervous	2.73	1.57	2.79	1.35	1.77	1.18	9.77	< 0.001	1	0.001	< 0.001
Tense	2.79	1.53	3.04	1.43	1.79	1.06	13.35	< 0.001	0.62	< 0.001	< 0.001
Calm	3.30	1.55	3.55	1.72	4.37	1.59	17.60	0.002	0.72	0.003	0.03
Indifferent	2.95	1.59	3.02	1.69	3.66	1.81	3.00	0.05	0.98	0.09	0.14
Neutral	3.23	1.75	3.61	1.89	4.48	1.71	7.27	< 0.001	0.54	0.001	0.04
Emotional	2.95	1.78	3.21	1.78	3.64	1.85	2.12	0.12	0.74	0.13	0.46
Alert	3.43	1.59	3.87	1.64	3.79	1.53	1.24	0.29	0.33	0.50	0.96
Aroused	2.07	1.26	2.45	1.55	2.12	1.18	1.29	0.28	0.34	0.98	0.45
Energetic	2.48	1.19	2.29	1.41	2.61	1.17	0.92	0.40	0.71	0.87	0.71
Excited	2.39	1.37	2.16	1.46	2.41	1.30	0.57	0.57	0.67	1	0.63

### The impact of state anxiety on false belief reasoning

Thirty-six participants’ percentages on the false belief task did not add up to 100%, varying from 90% to 330%, and therefore data was scaled for all participants’ responses as described in the method. One outlier was greater than 3 SDs from the mean and was therefore removed from analysis.

**Fig. 2:**
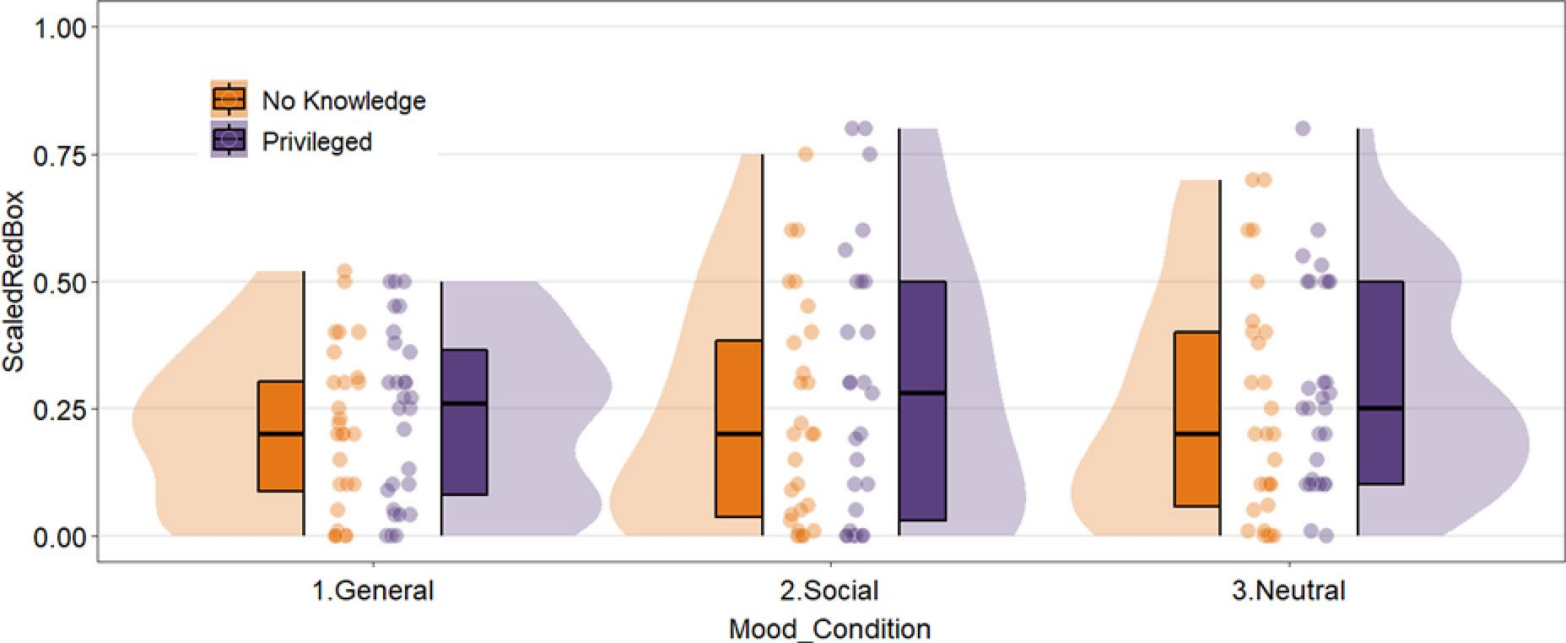
Violin plot of participants’ predictions that vicki will look in the red box by mood and knowledge condition.

A 3 (general anxiety, social anxiety, neutral) x 2 (privileged knowledge, no knowledge) between-subjects ANOVA yielded no main effect of mood condition, *F*(2, 161) = 0.88, *p* =.42, or knowledge condition, *F*(1, 161) = 2.08, *p* =.15, η² = 0.01 on participants’ prediction of how likely Vicki was to look in the red box. The interaction effect was also non-significant, *F*(5, 161) = 0.08, *p* =.92), η² < 0.01, see Fig. 2.

To confirm the results remained the same when controlling for trait general and social anxiety, an exploratory ANCOVA was conducted, following the same 3 × 2 design, with the addition of trait general and trait social anxiety as covariates. This ANCOVA found no main effect of mood condition, *F*(2, 161) = 0.1.47, *p* =.23, η² = 0.02, or knowledge condition, *F*(1, 161) = 1.47, *p* =.19, η² = 0.01 on participants’ prediction of how likely Vicki was to look in the red box. The interaction effect was also non-significant, *F*(5, 161) = 0.24, *p* =.79), η² < 0.01.

A sensitivity analysis was conducted to assess whether the findings were influenced by the effectiveness of the anxiety manipulation (see supplementary material, table S1). Results remained consistent after excluding participants whose self-rated anxiety contradicted their assigned condition and showed no significant correlation between anxiety ratings and theory of mind performance.

### Correlation between trait anxiety and false belief reasoning

Spearman’s correlations revealed a significant correlation between trait general and social anxiety in the no knowledge condition, *r* =.45, *p* <.001, but no significant correlations between trait general anxiety and scaled red box response *r* = −.03, *p* =.82, or trait social anxiety and scaled red box response, *r* =.21, *p* =.06. Similarly, for the privileged knowledge condition, there was a significant correlation between trait general and social anxiety, *r* =.39, *p* <.001, but no significant correlations between trait general anxiety and scaled red box response *r* = −.18, *p* =.11, or trait social anxiety and scaled red box response, *r* =.05, *p* =.64^1^.

## Discussion

In this study, participants’ state general and social anxiety was effectively manipulated after completing an autobiographical writing task, with a prospective future follow-up task. There was, however, no evidence that state general and social anxiety had an impact on theory of mind measured through a false belief task, when compared to a neutral mood group. There were also no significant correlations between trait general and social anxiety and theory of mind performance in the false belief task. There was a correlation between trait general and trait social anxiety, suggesting that people who experience trait general anxiety are more likely to also experience trait social anxiety and vice versa.

### The impact of state anxiety on theory of mind

The current study’s findings are inconsistent with previous studies on anxiety and theory of mind by Todd et al. (2015)^6^ and Todd and Simpson (2016)^35^. The findings from Todd et al. (2015)^6^ suggest that egocentrism is increased by emotions that are characterised by uncertainty. In the current study participants in each condition reported being significantly more anxious in the anxiety conditions, yet the outcomes did not demonstrate a motivation to reduce uncertainty, or this motivation may have been overridden. Todd and Simpson (2016)^35^ suggest that the impact of anxiety on theory of mind may be particularly noticeable for social aspects of cognition, however, the current study found no differences between social and non-social anxiety on a theory of mind task. Dyer et al. (2022)^36^, on the other hand, found no effects of state anxiety on emotion recognition, and although they did not distinguish between general and social anxiety, these findings are more consistent with the current study.

The discrepancy between the findings from this study and previous studies poses queries about whether anxious participants experienced increased egocentrism, and if they did, how this may not lead to a reduced theory of mind ability. This resonates with Todd and Tamir’s (2024)^21^ argument that the capability to override an egocentric pull depends on the strength with which egocentric information is activated, as well as individual characteristics that influence someone’s motivation or ability to override this pull. In the current study, the strength of the activation of self-information may rely on the intensity of experienced anxiety or the failure of the false belief task to create a curse of knowledge adequately, which is suggested from the absence of a significant main effect of knowledge state. Overall, the outcomes of this study indicate that even when people are anxious, they can still utilise their knowledge about others’ knowledge to make accurate, unbiased inferences in a false belief task.

### The impact of trait anxiety on theory of mind

Baez et al.’s (2023)^38^ meta-analysis looking at the impact of trait anxiety on theory of mind concludes that SAD produces a reduced theory of mind ability compared to healthy controls, despite some of the variability between studies. In the current study, trait social anxiety did not lead to a reduced theory of mind, which is more consistent with the findings from the study by Lenton-Brym et al. (2018)^43^ that found no differences between high and low socially anxious participants based on their scores from the SPIN. Considering that increased trait social anxiety was positively correlated with trait general anxiety in the current study, it may be worth considering if there are differences in the impact on theory of mind when these are present in isolation and in combination (i.e., trait social anxiety with no trait general anxiety, trait general anxiety with no trait social anxiety, and both trait social and general anxiety together).

While the non-significant findings of trait general and social anxiety in the current study echo the findings from Dyer et al. (2022)^36^, the latter study used an emotion recognition task. Emotion recognition is often associated with theory of mind^37^, but Quesque and Rossetti (2020)^45^ argue that emotion recognition tasks measure only a single component of theory of mind rather than the full construct. Despite this disjuncture, given the close association between these two concepts, these similarities in findings may suggest that the cognitive processes linking theory of mind and emotion recognition remain unaffected by trait general and social anxiety.

### Strengths and limitations

Previous studies that have looked at the influence of anxiety on theory of mind and its related concepts have failed to measure state and trait and general and social anxiety as distinct concepts. This study was the first to differentiate between both state and trait, and general and social anxiety, while using a well-established measure of theory of mind.

While the mood manipulation procedure was shown to adequately induce a mood state of sufficient intensity, i.e., participants in the anxiety conditions reported being significantly more anxious than the neutral group, it is not clear if the duration of this manipulated mood state was sufficient to affect the cognitive processes underlying theory of mind. There may also be a question around whether the curse of knowledge in the false belief task was sufficient to detect theory of mind deficits in this cohort of participants, as there was no effect of knowledge condition. Though in some ways surprising, the effect size was in line with more recent replications of the curse of knowledge effect, suggesting that though the effect is robust, it is likely smaller than reported in original studies^55^. It is also worth noting that the false belief task was not time limited. This may have allowed participants, including those experiencing anxiety, to compensate for initial egocentric responses. Time pressure can heighten cognitive load and may reveal more subtle impairments in perspective-taking, though it should be noted previous tasks have found an impact of anxiety without employing such a limit^6^. Additionally, some participants did not appear to fully understand the task instructions regarding percentages, as evidenced by entries that did not sum to 100. This may have introduced additional variability in the data and limits the extent to which responses can be interpreted as accurate reflections of participants’ judgments.

One possibility is that between participant variation on the Vicki’s violin task made it harder to demonstrate the manipulated effect of mood, within-subjects manipulations may be a useful alternative design. However, Converse et al. (2008)^22^ used similar mood manipulation procedures with moderate effect sizes and discovered significant differences in mood groups on Vicki’s Violin theory of mind task in a similar cohort of university students. Future work is needed to enhance our understanding of what different theory of mind tasks measure^7^ and determine which are best implemented for tracking the impact of state-level factors^21^. Similarly, all conditions in the task are inherently social in nature, so we have no way to determine whether our null result reflects participants not acting to reduce uncertainty, or egocentric responding not being consistent with such an approach in this case. For future study, including non-social control conditions may be important to disentangle these alternatives. Similarly, future work may look to differentiate between the impact of anxiety on egocentrism and more broadly on theory of mind performance.

The findings from this study are limited by the potentially mismatched impact of mood manipulation tasks within a laboratory environment when compared to real-world experiences in terms of (a) intensity and (b) differences between generalised and social anxiety. Bhanot et al. (2020)^56^ comment on the ecological validity of autobiographical mood induction tasks relative to other laboratory methods, noting that these can often encounter a demand effect due to the wording used and may be limited in external validity. Still, laboratory results have been evidenced to reliably replicate field results when there are large effect sizes^57,58^. Given that Converse et al. (2008)^22^ found a significant impact of mood on theory of mind with moderate effect sizes for manipulation check items, and the current study’s manipulation check demonstrated medium to large effect sizes, the non-significant effect in this study may indeed offer a reliable outcome of anxiety on theory of mind. It is also worth noting, however, that it is possible that the relationship between anxiety and theory of mind is particularly pronounced in contexts that are emotionally salient or socially threatening, such as interactions involving authority figures, romantic partners, or peers. The relatively neutral nature of the laboratory tasks may have lacked the interpersonal relevance necessary to elicit such effects, as suggested by findings showing that individuals with social anxiety disorder exhibit impaired theory of mind performance in socially evaluative contexts^39^.

Differences in general and social anxiety on the manipulation check items could help to ensure distinctions between the two conditions. That is, it could be argued that a participant’s anticipation of a larger audience for the social anxiety condition prospective follow-up task (having been told they would have to present their writing) could increase these distinctions. However, studies investigating the impact of audience size on level of social anxiety have found no significant effects^59,60^.

The current study showed good internal validity, given that there were no differences found between conditions in terms of participants’ age, gender, and ethnicity, suggesting that the non-significant findings are not a result of these factors. However, an overall majority of study participants identified as female. This imbalance potentially limits the generalisability of the findings to male samples, particularly as female participants have been shown to demonstrate a greater self-other distinction whilst under stress whereas male participants respond with increased egocentrism^61^. If women demonstrate an enhanced theory of mind ability under negatively valanced emotions, the majority female sample in the current study may have contributed to the absence of a significant main effect of anxiety on theory of mind.

### Future directions

The current study suggests several additional avenues for future research. First, future research should continue to determine which concepts are being measured (theory of mind, mentalizing, etc.) and when measuring theory of mind should use specific and well-established theory of mind tasks, such as the one used in this study. Equally, this may involve systematically testing different aspects of theory of mind to understand whether inconsistencies in findings might suggest truly different impacts of anxiety on different theory of mind sub-processes. They should also continue to distinguish systematically between state and trait, and general and social anxiety as distinct phenomena. Replicating the current study’s protocol could verify the findings. Additionally, future studies should ensure that participants fully understand task instructions, particularly when providing percentage-based responses, for example through practice trials or clearer guidance, to reduce data variability and improve interpretability. Second, further analyses could explore the relationship between trait anxiety and susceptibility to state mood manipulation. Third, while the current study did not incorporate integral emotions, similar distinctions between different emotional phenomena and reliable measures should be used when investigating how these may affect theory of mind. Finally, it may be worth considering situational factors that may induce anxiety more sufficiently and accurately when administering tasks, including stricter time constraints to increase the sensitivity of theory of mind tasks to anxiety-related effects. In addition, future studies should aim to recruit more balanced samples of gender and ethnicity so that outcomes can be generalised to broader populations and subgroup differences can be explored.

### Clinical implications

The significant, bi-directional correlation between trait general and trait social anxiety supports the conclusions from Goldenberg et al. (1996)^62^ that GAD and SAD are more commonly experienced as comorbidities than not. Despite this known association, quality standards for the treatment provided by the National Institute for Health and Care Excellence (NICE) for GAD and SAD differ; a stepped care approach is recommended for GAD and specifically developed Cognitive Behavioural Therapy (CBT) interventions are recommended for SAD^63^. Given the treatment distinctions, individuals presenting at mental health services with GAD or SAD should be screened for the presence of the other condition, and interventions should be offered for both conditions when necessary.

## Conclusions

The current study showed that state general and social anxiety did not impact the curse of knowledge people experience when reasoning about false beliefs. Equivalent null results were found for trait general and social anxiety. These findings prompt further questions about what factors may exist that provide someone with the ability to override an egocentric motivation to reduce uncertainty. The study builds upon previous research and begins to provide a systematic approach to discriminating between the varying impacts of these different phenomena on reliably measured theory of mind.

## Supplementary Information

Below is the link to the electronic supplementary material.

Supplementary Material 1

## Data Availability

SPSS data file that supports the findings of this study has been uploaded to Open Science Framework (OSF) project osf.io/zc3pf Registration DOI [https://doi.org/10.17605/OSF.IO/4KA9Y].

## Footnotes

1 An exploratory linear regression analysis showed that an interaction term between GAD-7 score and SPIN score did not predict performance on the privileged knowledge condition either.

## References

[1] Lin, S., Keysar, B. & Epley, N. Reflexively Mindblind: using theory of Mind to interpret behavior requires effortful attention. *J. Exp. Soc. Psychol.* **46**, 551–556 (2010).

[2] Remes, O., Brayne, C., van der Linde, R. & Lafortune, L. A systematic review of reviews on the prevalence of anxiety disorders in adult populations. *Brain Behav.* **6** (7), e00497. 10.1002/brb3.497 (2016). 27458547 10.1002/brb3.497 PMC4951626

[3] Crosier, B. S., Webster, G. D. & Dillon, H. M. Wired to connect: evolutionary psychology and social networks. *Rev. Gen. Psychol.* **16**, 230–239 (2012).

[4] Li, J. Humans as social beings—From ‘people first’ to people-centered. *Sci. Soc. Res.* **2**, Article2 (2020).

[5] Mühl, J. Human beings as social beings: Gerda walther’s anthropological approach. In Gerda Walther’s Phenomenology of Sociality, Psychology, and Religion (ed. 71–84 (Springer International Publishing, (2018).

[6] Todd, A. R., Forstmann, M., Burgmer, P., Brooks, A. W. & Galinsky, A. D. Anxious and egocentric: how specific emotions influence perspective taking. *J. Exp. Psychol. Gen.* **144**, 374–391 (2015). 25602753 10.1037/xge0000048

[7] Quesque, F. et al. Defining key concepts for mental state attribution. *Commun. Psychol.* **2**, 1–5 (2024). 39242813 10.1038/s44271-024-00077-6 PMC11332223

[8] Rusch, T., Steixner-Kumar, S., Doshi, P., Spezio, M., & Gläscher, J. Theory of mind and decision science: Towards a typology of tasks and computational models. *Neuropsychologia* **146**, 107488 (2020). 10.1016/j.neuropsychologia.2020.107488 32407906

[9] Yeung, E. K. L., Apperly, I. A. & Devine, R. T. Measures of individual differences in adult theory of mind: A systematic review. *Neuroscience Biobehavioral Reviews* **157** (2024). 10.1016/j.neubiorev.2023.105481 38036161

[10] Briscoe, H., Vickers-Graver, B., Jones, C. M. & Surtees, A. C. The link between anxiety and theory ofmind in children: A meta-analysis. *J. Affect. Disord.* **367**, 530–544 (2024). 10.1016/j.jad.2024.08.171 39214373

[11] Wimmer, H. & Perner, J. Beliefs about beliefs: representation and constraining function of wrong beliefs in young children’s Understanding of deception. *Cognition* **13**, 103–128 (1983). 6681741 10.1016/0010-0277(83)90004-5

[12] Birch, S. A. J. & Bloom, P. The curse of knowledge in reasoning about false beliefs. *Psychol. Sci.* **18**, 382–386 (2007). 17576275 10.1111/j.1467-9280.2007.01909.x

[13] Baron-Cohen, S., O’Riordan, M., Stone, V., Jones, R. & Plaisted, K. Recognition of faux Pas by normally developing children and children with asperger syndrome or high-functioning autism. *J. Autism Dev. Disord.* **29**, 407–418 (1999). 10587887 10.1023/a:1023035012436

[14] Happé, F. G. E. An advanced test of theory of mind: Understanding of story characters’ thoughts and feelings by able autistic, mentally handicapped, and normal children and adults. *J. Autism Dev. Disord*. **24**, 129–154 (1994). 8040158 10.1007/BF02172093

[15] Baron-Cohen, S., Wheelwright, S., Hill, J., Raste, Y. & Plumb, I. The reading the Mind in the eyes test revised version: a study with normal adults, and adults with asperger syndrome or high-functioning autism. *J. Child Psychol. Psychiatry Allied Discip.* **42**, 241–251 (2001). 11280420

[16] Povinelli, D. J. & Preuss, T. M. Theory of mind: evolutionary history of a cognitive specialization. *Trends Neurosci.* **18**, 418–424 (1995). 7482808 10.1016/0166-2236(95)93939-u

[17] Henry, J. D., Phillips, L. H., Ruffman, T. & Bailey, P. E. A meta-analytic review of age differences in theory of Mind. *Psychol. Aging*. **28**, 826–839 (2013). 23276217 10.1037/a0030677

[18] Wu, S., Barr, D., Gann, T. & Keysar, B. How culture influences perspective taking: differences in correction, not integration. *Front Hum. Neurosci.* **7** (2013). 10.3389/fnhum.2013.00822 PMC3845341 24348368

[19] Cutting, A. L. & Dunn, J. Theory of mind, emotion understanding, language, and family background: individual differences and interrelations. *Child. Dev.* **70**, 853–865 (1999). 10446724 10.1111/1467-8624.00061

[20] Peterson, C. C. & Slaughter, V. P. Telling the story of theory of mind: deaf and hearing children’s narratives and mental state Understanding. *Br. J. Dev. Psychol.* **24**, 151–179 (2006).

[21] Todd, A. R. & Tamir, D. I. Factors that amplify and attenuate egocentric mentalizing. *Nat. Rev. Psychol.* **3**, 164–180 (2024).

[22] Converse, B. A., Lin, S. & Keysar, B. Epley, N. In the mood to get over yourself: mood affects theory-of-mind use. *Emotion* **8**, 725–730 (2008). 18837624 10.1037/a0013283

[23] Yip, J. A. & Schweitzer, M. E. Losing your temper and your perspective: anger reduces perspective-taking. *Organ. Behav. Hum. Decis. Process.* **150**, 28–45 (2019).

[24] Lerner, J. S., Li, Y., Valdesolo, P. & Kassam, K. S. Emotion and decision making. *Annu. Rev. Psychol.* **66**, 799–823 (2015). 25251484 10.1146/annurev-psych-010213-115043

[25] Raghunathan, R. & Pham, M. T. All negative moods are not equal: motivational influences of anxiety and sadness on decision making. *Organ. Behav. Hum. Decis. Process.* **79**, 56–77 (1999). 10388609 10.1006/obhd.1999.2838

[26] Eysenck, M., Payne, S. & Santos, R. Anxiety and depression: Past, present, and future events. *Cogn. Emot.* **20** (2), 274–294. 10.1080/02699930500220066 (2006).

[27] Stein, D. J. & Nesse, R. M. Threat detection, precautionary responses, and anxiety disorders. *Neurosci. Biobehav Rev.* **35**, 1075–1079 (2011). 21147162 10.1016/j.neubiorev.2010.11.012

[28] Gino, F., Brooks, A. W. & Schweitzer, M. E. Anxiety, advice, and the ability to discern: feeling anxious motivates individuals to seek and use advice. *J. Pers. Soc. Psychol.* **102**, 497–512 (2012). 22121890 10.1037/a0026413

[29] Shankman, S. A. & Klein, D. N. The relation between depression and anxiety: an evaluation of the tripartite, approach-withdrawal and valence-arousal models. *Clin. Psychol. Rev.* **23**, 605–637 (2003). 12788112 10.1016/s0272-7358(03)00038-2

[30] Khdour, H. Y. et al. Generalized anxiety disorder and social anxiety disorder, but not panic anxiety disorder, are associated with higher sensitivity to learning from negative feedback: behavioral and computational investigation. *Front. Integr. Neurosci.* **10**, 20 (2016). 27445719 10.3389/fnint.2016.00020 PMC4925696

[31] Yang, Y. et al. Cognitive impairment in generalized anxiety disorder revealed by event-related potential N270. *Neuropsychiatr Dis. Treat.* **11**, 1405–1411 (2015). 26082637 10.2147/NDT.S84666 PMC4461089

[32] Surtees, A. D. R., Briscoe, H. & Todd, A. R. Anxiety and mentalizing: uncertainty as a driver of egocentrism. *Curr. Dir. Psychol. Sci.* **33**, 100–107 (2024).

[33] Goldin, P. R., Manber, T., Hakim, S., Canli, T. & Gross, J. J. Neural bases of social anxiety disorder: emotional reactivity and cognitive regulation during social and physical threat. *Arch. Gen. Psychiatry*. **66**, 170–180 (2009). 19188539 10.1001/archgenpsychiatry.2008.525 PMC4142809

[34] Newman, M. G., Llera, S. J., Erickson, T. M., Przeworski, A. & Castonguay, L. G. Worry and generalized anxiety disorder: a review and theoretical synthesis of evidence on nature, etiology, mechanisms, and treatment. *Annu. Rev. Clin. Psychol.* **9**, 275–297 (2013). 23537486 10.1146/annurev-clinpsy-050212-185544 PMC4964851

[35] Todd, A. R. & Simpson, A. J. Anxiety impairs spontaneous perspective calculation: evidence from a level-1 visual perspective-taking task. *Cognition* **156**, 88–94 (2016). 27522111 10.1016/j.cognition.2016.08.004

[36] Dyer, M. L., Attwood, A. S., Penton-Voak, I. S. & Munafò, M. R. The role of state and trait anxiety in the processing of facial expressions of emotion. *R Soc. Open. Sci.* **9**, 210056 (2021). 10.1098/rsos.210056 PMC8728173 35070339

[37] Mier, D. et al. The involvement of emotion recognition in affective theory of Mind. *Psychophysiology* **47** (6), 1028–1039. 10.1111/j.1469-8986.2010.01031 (2010). 20456660 10.1111/j.1469-8986.2010.01031.x

[38] Baez, S., Tangarife, M. A., Davila-Mejia, G., Trujillo-Güiza, M., & Forero, D. A. Performance in emotion recognition and theory of mind tasks in social anxiety and generalized anxiety disorders: a systematic review and meta-analysis. *Front. Psychiatry* **14**, 1192683 (2023). 10.3389/fpsyt.2023.1192683 PMC10235477 37275989

[39] Hezel, D. M. & McNally, R. J. Theory of Mind impairments in social anxiety disorder. *Behav. Ther.* **45**, 530–540 (2014). 24912465 10.1016/j.beth.2014.02.010

[40] Washburn, D., Wilson, G., Roes, M., Rnic, K. & Harkness, K. L. Theory of Mind in social anxiety disorder, depression, and comorbid conditions. *J. Anxiety Disord*. **37**, 71–77 (2016). 26658117 10.1016/j.janxdis.2015.11.004

[41] Ballespí, S., Vives, J., Sharp, C. & Tobar, A. Barrantes-Vidal, N. Hypermentalizing in social anxiety: evidence for a context-dependent relationship. *Front. Psychol.* **10**, 1501 (2019). 31354562 10.3389/fpsyg.2019.01501 PMC6629962

[42] Alvi, T., Kouros, C. D., Lee, J., Fulford, D. & Tabak, B. A. Social anxiety is negatively associated with theory of Mind and empathic accuracy. *J. Abnorm. Psychol.* **129**, 108–113 (2020). 31697138 10.1037/abn0000493

[43] Lenton-Brym, A. P., Moscovitch, D. A., Vidovic, V., Nilsen, E. & Friedman, O. Theory of Mind ability in high socially anxious individuals. *Anxiety Stress Coping*. **31**, 487–499 (2018). 29940803 10.1080/10615806.2018.1483021

[44] Zainal, N. H. & Newman, M. G. Worry amplifies theory-of-mind reasoning for negatively valenced social stimuli in generalized anxiety disorder. *J. Affect. Disord*. **227**, 824–833 (2018). 29254067 10.1016/j.jad.2017.11.084 PMC6707505

[45] Quesque, F. & Rossetti, Y. What do theory-of-mind tasks actually measure? Theory and practice. *Perspect. Psychol. Sci.* **15**, 384–396 (2020). 32069168 10.1177/1745691619896607

[46] Spitzer, R. L., Kroenke, K., Williams, J. B. W. & Löwe, B. A brief measure for assessing generalized anxiety disorder: the GAD-7. *Arch. Intern. Med.* **166**, 1092–1097 (2006). 16717171 10.1001/archinte.166.10.1092

[47] Connor, K. M. et al. Psychometric properties of the social phobia inventory (SPIN): new self-rating scale. *Br. J. Psychiatry*. **176**, 379–386 (2000). 10827888 10.1192/bjp.176.4.379

[48] Ballance, B. C. et al. Imagining emotional events benefits future-oriented decisions. *Q J. Exp. Psychol* **17470218221086637**, (2022). 10.1177/17470218221086637 PMC9619259 35225089

[49] Endler, N. S. & Kocovski, N. L. State and trait anxiety revisited. *J. Anxiety Disord*. **15**, 231–245 (2001). 11442141 10.1016/s0887-6185(01)00060-3

[50] Grupe, D. W. & Nitschke, J. B. Uncertainty and anticipation in anxiety. *Nat. Rev. Neurosci.* **14**, 488–501 (2013). 23783199 10.1038/nrn3524 PMC4276319

[51] Miloyan, B., Pachana, N. A. & Suddendorf, T. Future-oriented thought patterns associated with anxiety and depression in later life: the intriguing prospects of prospection. *Gerontologist* **57**, 619–625 (2017). 26874188 10.1093/geront/gnv695 PMC5881767

[52] Damasio, A. R. et al. Subcortical and cortical brain activity during the feeling of self-generated emotions. *Nat. Neurosci.* **3**, 1049–1056 (2000). 11017179 10.1038/79871

[53] Blanca, M. J., Alarcón, R., Arnau, J., Bono, R. & Bendayan, R. Non-normal data: Is ANOVA still a valid option? *Psicothema* **29**, 552–557 (2017). 10.7334/psicothema2016.383 29048317

[54] Ghasemi, A. & Zahediasl, S. Normality tests for statistical analysis: A guide for non-statisticians. *Int. J. Endocrinol. Metab.* **10**, 486–489 (2012). 23843808 10.5812/ijem.3505 PMC3693611

[55] Ryskin, R. A. & Brown-Schmidt, S. Do adults show a curse of knowledge in false-belief reasoning? A robust estimate of the true effect size. *PLOS ONE*, **9**, e92406 (2014). 10.1371/journal.pone.0092406 PMC3965426 24667826

[56] Bhanot, S. P., Chang, D., Cunningham, J. L. & Ranson, M. Emotions and decisions in the real world: what can we learn from quasi-field experiments? *PLOS ONE*. **15**, e0243044 (2020). 33326430 10.1371/journal.pone.0243044 PMC7744061

[57] Anderson, C. A., Lindsay, J. J. & Bushman, B. J. Research in the psychological laboratory: truth or triviality? *Curr Dir. Psychol. Sci.* **8**, 3–9 (1999).

[58] Mitchell, G. Revisiting truth or triviality: the external validity of research in the psychological laboratory. *Perspect. Psychol. Sci.* **7**, 109–117 (2012). 26168439 10.1177/1745691611432343

[59] Mostajeran, F., Balci, M. B., Steinicke, F., Kühn, S. & Gallinat, J. The effects of virtual audience size on social anxiety during public speaking. *2020 IEEE Conference on Virtual Reality and 3D User Interfaces (VR)* 303–312 (2020).

[60] Wankel, L. M. Audience size and trait anxiety effects upon state anxiety and motor performance. *Res. Q. Am. Alliance Health Phys. Educ. Recreat*. **48**, 181–186 (1977). 10.1080/10671315.1977.10762168 266235

[61] Tomova, L., von Dawans, B., Heinrichs, M., Silani, G. & Lamm, C. Is stress affecting our ability to tune into others? Evidence for gender differences in the effects of stress on self-other distinction. *Psychoneuroendocrinology* **43**, 95–104 (2014). 24703175 10.1016/j.psyneuen.2014.02.006

[62] Goldenberg, I. M. et al. The infrequency of. *J. Clin. Psychiatry*. **57**, 9093 (1996). 10.4088/jcp.v57n1105 8968302

[63] National Institute for Health and Care Excellence. Psychological interventions: Anxiety disorders [NICE Quality Standards No53]. (2014).

