# Supplementary material for: The impact of state and trait general and social anxiety on theory of mind
Source: Sci Rep. 2026 Mar 5;16:8232. doi: 10.1038/s41598-026-36718-5 (PMC12963424; doi:10.1038/s41598-026-36718-5)
Supplement: Supplementary file 1 — Supplementary Material 1 [file 41598_2026_36718_MOESM1_ESM.docx]

**Supplementary Materials**

**Bootstrapping**

The dependent False Belief variable was not normally distributed. Although ANOVAs are typically thought to be robust to such deviation, given appropriate sample sizes, we conducted further analysis to confirm that the core analysis were not unduly impacted. This involved employing bootstrapping. A bootstrapped 3 (general anxiety, social anxiety, neutral) x 2 (privileged knowledge, no knowledge) between-subjects ANOVA with 1000 samples yielded no main effect of mood condition, *F*(2, 161) = .88, *p* = .42, η² = .01, or knowledge condition, *F*(1, 161) = 2.08, *p* = .15, η² = .01, on participants’ prediction of how likely Vicki was to look in the red box. The interaction effect was also non-significant, *F*(5, 161) = .08, *p* = .92), η² < .01

**Sensitivity Analysis for Effectiveness of Manipulation Check**

Further analyses were conducted to check whether the core finding was influenced by the effectiveness of the anxiety manipulation. Given different participants may use scales differently, there was no objective measure of whether individual participants were anxious. Two alternative approaches were therefore undertaken. Firstly, participants in the anxiety conditions who rated their anxiety below the mean of the relaxed condition (1.82) and participants in the relaxed condition who rated their anxiety above the mean of the anxiety conditions (2.97) were removed. This led to nine participants being removed from the general anxiety condition, five from the social anxiety condition and 12 from the relaxed condition. The subsequent 2 x 3 ANOVA examining the impact of Condition and Knowledge State on performance found no effect of Condition, *F*(2, 141) = .18, *p* = .84, no effect of Knowledge State, *F*(1, 141) = 1.65, *p* = .20, and no interaction between the two , *F*(2, 141) = .20, *p* = .82. This suggested no difference from the findings without removing these participants. Secondly, we considered whether there was a correlation between self-rated anxiety and likelihood of looking in the red box (independent) of condition, for those participants with a privileged knowledge stage. No significant corelation was found, ρ(83) = -.094, *p =* .4.

**Supplementary Table S1**

*Tests of Between-Subjects Effects^[[1]](#footnote-1)^*

*Dependent Variable: Scaled percentage*

| Source | Type III Sum of Squares | df | Mean Square | F | Sig. | Partial Eta Squared |
| --- | --- | --- | --- | --- | --- | --- |
| Corrected Model | .104^[[2]](#footnote-2)^ | 5 | .021 | .462 | .804 | .017 |
| Intercept | 9.244 | 1 | 9.244 | 205.946 | <.001 | .604 |
| Stateconditions | .016 | 2 | .008 | .179 | .836 | .003 |
| Taskcondition_n | .074 | 1 | .074 | 1.653 | .201 | .012 |
| Stateconditions * taskcondition_n | .018 | 2 | .009 | .197 | .821 | .003 |
| Error | 6.060 | 135 | .045 |  |  |  |
| Total | 15.387 | 141 |  |  |  |  |
| Corrected Total | 6.163 | 140 |  |  |  |  |

1. Sensitivity = . [↑](#footnote-ref-1)
2. R Squared = .017 (Adjusted R Squared = -.020) [↑](#footnote-ref-2)
